# Genetic structure of six cattle populations revealed by transcriptome-wide SNPs and gene expression

**DOI:** 10.1007/s13258-018-0677-1

**Published:** 2018-03-26

**Authors:** Wei Wang, Huai Wang, Hui Tang, Jia Gan, Changgeng Shi, Qing Lu, Donghui Fang, Jun Yi, Maozhong Fu

**Affiliations:** 1 Sichuan Animal Science Academy, Animal Breeding and Genetics Key Laboratory of Sichuan Province, Chengdu, 610066 Sichuan People’s Republic of China; 2 Xuanhan Animal Breeding and Improvement Station, Xuanhan County, 636150 Sichuan People’s Republic of China; 3 Sichuan Yangping Cow Breeding Farm, Hongya County, 620360 Sichuan People’s Republic of China

**Keywords:** Cattle, RNA-Seq, SNPs, Gene expression, Population structure

## Abstract

There are abundant cattle breeds/populations in China, and the systematic discovery of genomic variants is essential for performing the marker assisted selection and conservation of genetic resources. In the present study, we employed whole transcriptome sequencing (RNA-Seq) technology for revealing genetic structure among six Chinese cattle populations according to transcriptome-wide SNPs and gene expression. A total of 68,094 variants consisting of 61,754 SNPs and 6340 InDels were detected and widely distributed among all chromosomes, by which the clear patterns of population structures were revealed. We also found the significantly differential density of variant distribution among genes. Additionally, we totally assembled 15,992 genes and detected obvious differences on the expression profiles among populations. In contrast to genomic variants, the measure of gene expression levels failed to support the expected population structure. Here, we provided a global landscape on the differential expression genes among these cattle populations.

**Electronic supplementary material:**

The online version of this article (10.1007/s13258-018-0677-1) contains supplementary material, which is available to authorized users.

## Introduction

Due to rapid advances of the cost-efficient and high-throughput sequencing technology, whole transcriptome sequencing (RNA-Seq) has become a common strategy in biological and medical researches (Wang et al. 2009; Finotello and Di Camillo 2015). RNA-Seq technology has been well known to be preferable to the traditional DNA microarray approach for quantifying gene expression, especially in term of sensitivity. Actually, RNA-Seq is also powerful for de novo explore nucleotide variants being exclusively derived these transcribed regions, such as single nucleotide polymorphism (SNP) (Chepelev et al. 2009). Therefore, the RNA-Seq would be very helpful in studies of non-model organisms in absence of reference genome and annotation information (Ekblom and Galindo 2011). Certainly, it is also expected that RNA-Seq will be employed more prevalent into studies of population genetics due to the ongoing decrease of sequencing cost and development of simple bioinformatic pipelines (De Wit et al. 2012).

Modern cattle (*Bos taurus*) is one of the most important domesticated animals with tremendous contribution to human civilization. Now, > 800 domestic cattle breeds have been recognized with various morphological characters (Lewis et al. 2011). Among them, however, only a few breeds are absolutely predominant and widely reared throughout world due to outstanding production performances, such as Holstein, Simmental, Hereford and Angus. Of course, there is a large amount of excellent genetic resources also reserved in the indigenous cattle breeds, which have only been distributed within the restricted areas with lower performances for economic traits of interest. By artificial breeding program, these excellent genetic resources could be employed for further improving production performances in future (Tixier-Boichard et al. 2015). In China, there are a large number of indigenous cattle breeds with the increasing demands for genetic selection and improvements (Jia 2004). However, genetic structure for Chinese indigenous cattle has largely remained unknown.

In addition to gene expression profiling, RNA-Seq has increasingly been used for scanning genomic variants in human (Piskol et al. 2013), wild and domestic animals (Schunter et al. 2014; Wickramasinghe et al. 2014; Konczal et al. 2014). In cattle, Cánovas et al. systematically investigated the transcriptome-wide SNPs among milk samples of Holstein cows by RNA-Seq and further provided a practical guidance to improve accuracy of SNP discovery (Cánovas et al. 2010). Furthermore, individual blastocyst of Holstein was also sequenced by RNA-Seq for gene expression profiling and SNP discovery (Chitwood et al. 2013). However, RNA-Seq has rarely been used for study of population genetics in cattle yet. In the present study, therefore, we employed RNA-Seq technology and comprehensively investigated transcriptome-wide variants and gene expression among six cattle populations in Sichuan, China; and by which the genetic diversity and structure were analyzed. All of these collected populations could be classified between indigenous and imported breeds. The results would provide helpful clues for better establishing artificial breeding schemes for these indigenous cattle.

## Materials and methods

### Sampling and genetic relationships of populations

A total of 29 whole blood samples were collected from the 12-month-old healthy cattle, which consists of six breeds and populations of Simmental bull (SM, N = 5), Holstein heifer (HS, N = 5), Xuanhan bull (XH, N = 4), F1 generations heifer (F1, N = 5) of XH and SM, F2 generations heifer (F2, N = 5) of XH, SM and HS, and Shuxuan bull (SX, N = 5). Both SM and HS are the foreign breeds, while XH is an indigenous cattle breed in Sichuan. The others are the cultivated breeds (SX) or immediate populations (F1 and F2). These cattle have been simultaneously reared in a commercial farm in Sichuan and therefore subjected to the same feeding and management procedure. The genetic relationships among six populations are shown in Supplementary Fig. 1.

### RNA extraction and sequencing

The whole blood samples were immediately nap-frozen in liquid nitrogen for total RNA extraction, for which the TRIzol reagent (Invitrogen, Shanghai, China) was used according to manufacturer’s instructions. After samples were treated by DNase, concentration and quality of total RNA were evaluated using Agilent 2100 Bioanalyser (Agilent, Santa Clara, USA); and only samples with high RNA quality were finally employed. Subsequently, mRNA sequencing libraries were prepared using RNA-Seq Sample Preparation Kit (Illumina, San Diego, USA) according to official instruction. In brief, the poly(A) mRNA was isolated by poly-dT bead from total RNA and chemically fragmented to approximately 200 bp fragments. After two-strand cDNA was synthesized, DNA fragments were selected by fragment size and then amplified by PCR. The amplified mRNA libraries with the expected size of 200 bp were sequenced on Illumina HiSeq™ 2000 platform for generating 90 bp paired-end reads.

### Quality filtering of reads

The initial images from sequencer were first converted into sequence files in fastq format according to official pipeline. For the raw reads, we subsequently conducted quality filtering and discarded these low quality reads that are defined as one of the following types: (i) read containing > 50% bases with the quality values below 14, (ii) read containing > 2% unambiguous bases, or (iii) reads containing adaptor sequences. This step was conducted using tool of NGS QC Toolkit (Patel and Jain 2012), and after which we got clean reads.

### Reads mapping and variant calling

We employed GATK toolkit for calling transcriptome-wide variants from RNA-Seq reads (McKenna et al. 2010). First, clean reads were mapped to reference genome of cattle retrieved from Ensembl (UMD3.1.81) using BWA tool (Li and Durbin 2009), in which the BWA-MEM algorithm was used with default parameters. After getting raw alignment files in BAM format, we further restricted the variant calling within coding regions; and this effort was expected to improve accuracy of variants. Subsequently, we performed standard Picard-GATK pipeline to get raw variants using Picard 2.0.1 (http://picard.sourceforge.net/) and GATK toolkit 3.5 (McKenna et al. 2010).

All sample-wise raw variants were combined together for producing whole profile of variants among 29 samples using HaplotypeCaller module of GATK (McKenna et al. 2010). The quality filtering was subsequently applied to all variants according to the recommended standard (including values of QD < 2.0, FS > 60.0, MQ < 40, MappingQualityRankSum < 10.5, HaplotypeScore > 13.0 and ReadPosRankSum < − 8.0). In addition, we further performed custom filtering steps for guaranteeing rigorous quality control, including removals of low-coverage variants (average coverage < 5) and variants with minor allele frequency < 0.05. Here, we obtained high quality variants for further analysis.

### Transcript assembly and quantification

In the present study, we employed the genome-guided method for transcriptome assembly. Briefly, clean reads were mapped against reference genome of cattle using Tophat v2.0.9 (Kim et al. 2013) with the following parameters: maximum mismatches per read of 3, maximum edit distance per read of 4, maximum edit distance to re-align per read of 2, and other parameters by defaults. We subsequently assembled transcripts by cufflinks v2.2.0 (Trapnell et al. 2012) in aid of the reference annotation (UMD3.1.81). Based on the assembled transcriptome, we remapped all clean reads and finally quantified gene expression using RSEM (Li and Dewey 2011); in which the rsem-calculate-expression module was used for producing measures of the estimated count of fragments (ECF) of gene.

### Population demography based on transcriptome-wide SNPs and gene expressions

Because most of the detected variants were SNPs with little proportion of InDels, we only employed SNPs for dissecting population demography. First, the pair-wise Euclidean distances among all samples were calculated according to clean SNPs by SNPRelate R package (Zheng et al. 2012). Second, the calculated dissimilarities matrix was subjected to both principal coordinate analysis using Stat R packages and hierarchical clustering for revealing genetic relationships. Finally, the fixation indexes (Fst) were estimated among six cattle breeds for evaluating population differentiation and genetic relatedness using SNPRelate (Zheng et al. 2012).

The raw ECF values of gene expressions were first normalized by TMM method implemented in Edge R package (Robinson et al. 2010). After the log transformation, we employed the LIMMA R package (Ritchie et al. 2015) for performing differential expression (DE) of genes according to linear model:

$${y_{ij}}=\mu +{b_i}+{\varepsilon _{ij}},$$

where ${y_{ij}}$ is the $j{\text{th}}$ observation in $i{\text{th}}$ breed, $\mu$ the overall mean of expression level, ${b_i}$ the mean of expression level in $i{\text{th}}$ breed, and ${\varepsilon _{ij}}$ the residual errors.

We first analyzed the overall DE genes among six populations by the moderated F-statistic with Benjamini–Hochberg (BH) adjustment for multiple testing. Based on DE genes, we estimated the genetic relationships among six populations and further conducted the bi-clustering for both genes and populations to investigate potential substructure. Finally, pair-wise comparisons were also conducted for revealing DE genes for providing more insights into genetic similarity at population level.

## Results

### Transcriptome-wide profiling of variants

A total of ~ 827 million raw paired reads were generated with 100 bp in length. After quality filtering, an average of 25.82 million paired reads was finally obtained for each sample (Table 1). Within the annotated coding region of protein-encoding genes, which accounts for 1.58% of whole genome in length, we successfully detected 246,603 raw variants consisting of SNP and InDel. We subsequently performed strict quality filtering and finally obtained 68,094 clean variants among all 29 samples, including 61,754 SNPs and 6340 InDels (Table 1).

**Table 1:** Means of sequenced reads and variants within each population

Populations	Raw paired reads	Clean paired reads	SNPs	InDels
Simmental (SM)	28,959,868	26,059,878	17,458	3456
Holstein (HS)	26,592,017	24,442,859	16,788	3248
Xuanhan (XH)	27,974,983	24,776,862	30,981	3830
F1	33,041,548	30,068,077	27,538	3740
F2	27,057,105	24,350,083	21,147	3345
Shuxuan (SX)	27,365,491	24,987,881	23,212	3623
Total	28,516,554	25,815,563	61,754	6340

**Fig. 1:**
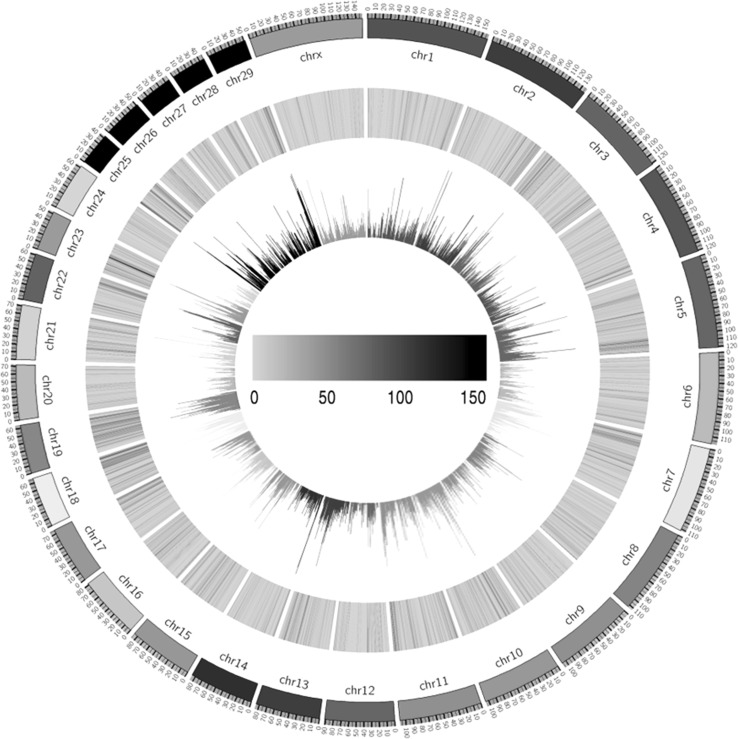
Circos plot shows the genome-wide distribution density of all clean variants. The outer ring represents ideograms of 29 autosomal and X chromosomes. Density of variants distributed along with chromosomes are demonstrated by the inner ring of bar charts. Meanwhile, the density of exon regions, within which the variants were scanned, are also shown by the intermediate ring

**Fig. 2:**
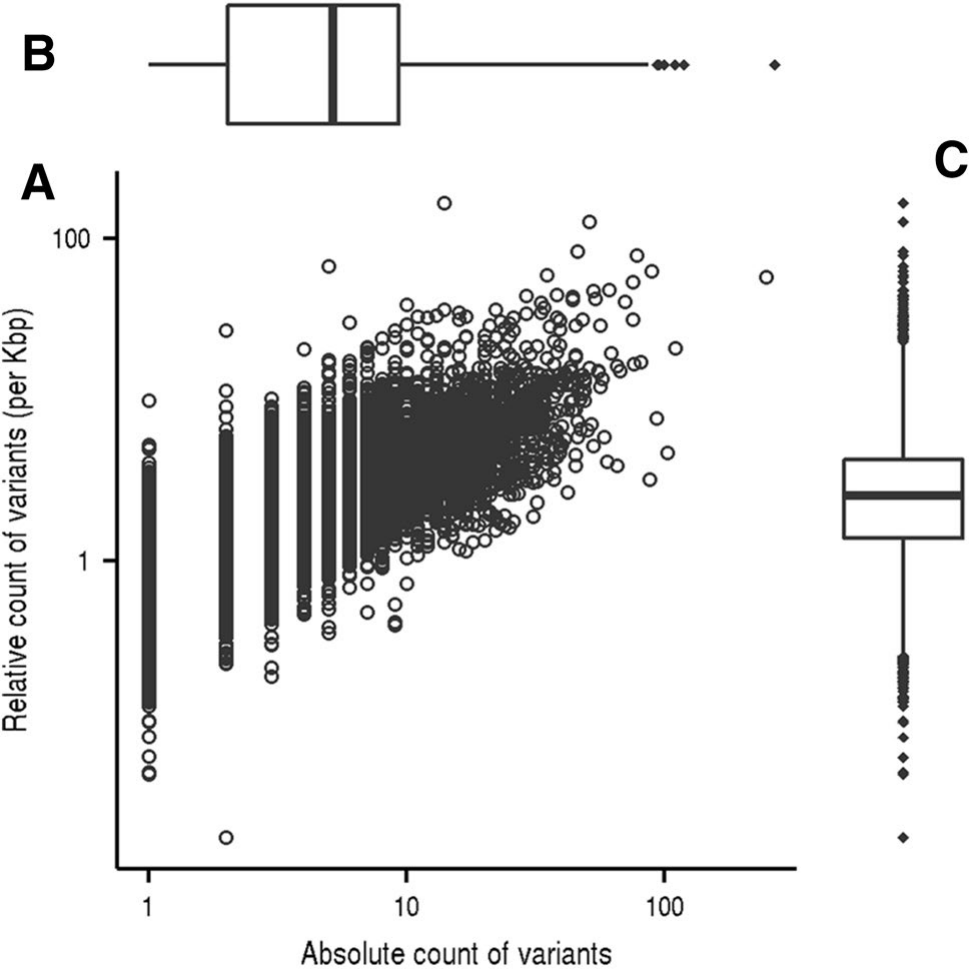
Gene-wise comparisons of variant distribution. **a** Dot plot demonstrates the correlation between absolute counts and relative counts (per kilobase of exon) of variants within each gene. Box-whisker plots further show the overall distributions of absolute (**b**) and relative (**c**) counts of variants, in which the box extends from the 25th to 75th percentiles with median value of middle lines

The clean variants were proportionally distributed among all 29 autosomal and X chromosomes (Fig. 1). On average, three variants were detected per kilobase exons. Furthermore, intra-gene comparisons of variant distribution showed that there was a slight correlation between the absolute and relative counts of variants per gene; and the latter was defined as count of variants per kilobase of exon in length. However, there were considerable inter-gene variations for both the absolute and relative counts of variants (Fig. 2). The gene-wise differences on genetic polymorphism would suggest differential importance on the biological functions and/or evolutionary roles. Therefore, we herein listed the top 50 genes having the highest density of variant distribution (Supplementary Table 1), which has mean of 38.56 variants per kilobase exon.

### Population demography based on SNPs

**Fig. 3:**
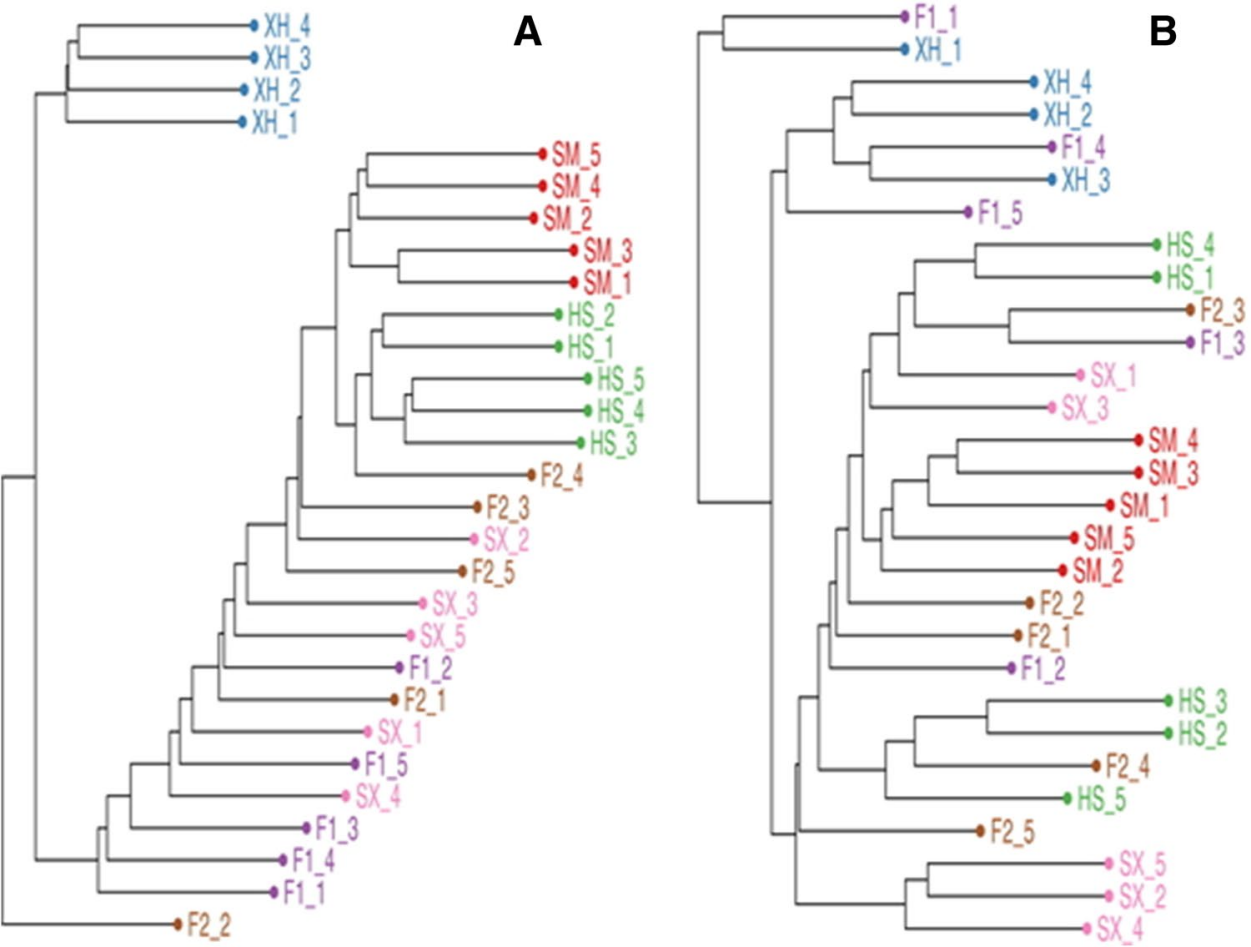
Hierarchical clustering trees of 29 samples based on transcriptome-wide SNPs (**a**) and gene expression (**b**). Two letter abbreviations of ‘SM’ for Simmental, ‘HS’ for Holstein, ‘XH’ for Xuanhan, ‘F1’ for F1 generation, ‘F2’ for F2 generation, and ‘SX’ for Shuxuan cattle were used throughout all figures if necessary. All samples from each population (marked by different colours) were further suffixed by the sequential numbers. (Color figure online)

**Fig. 4:**
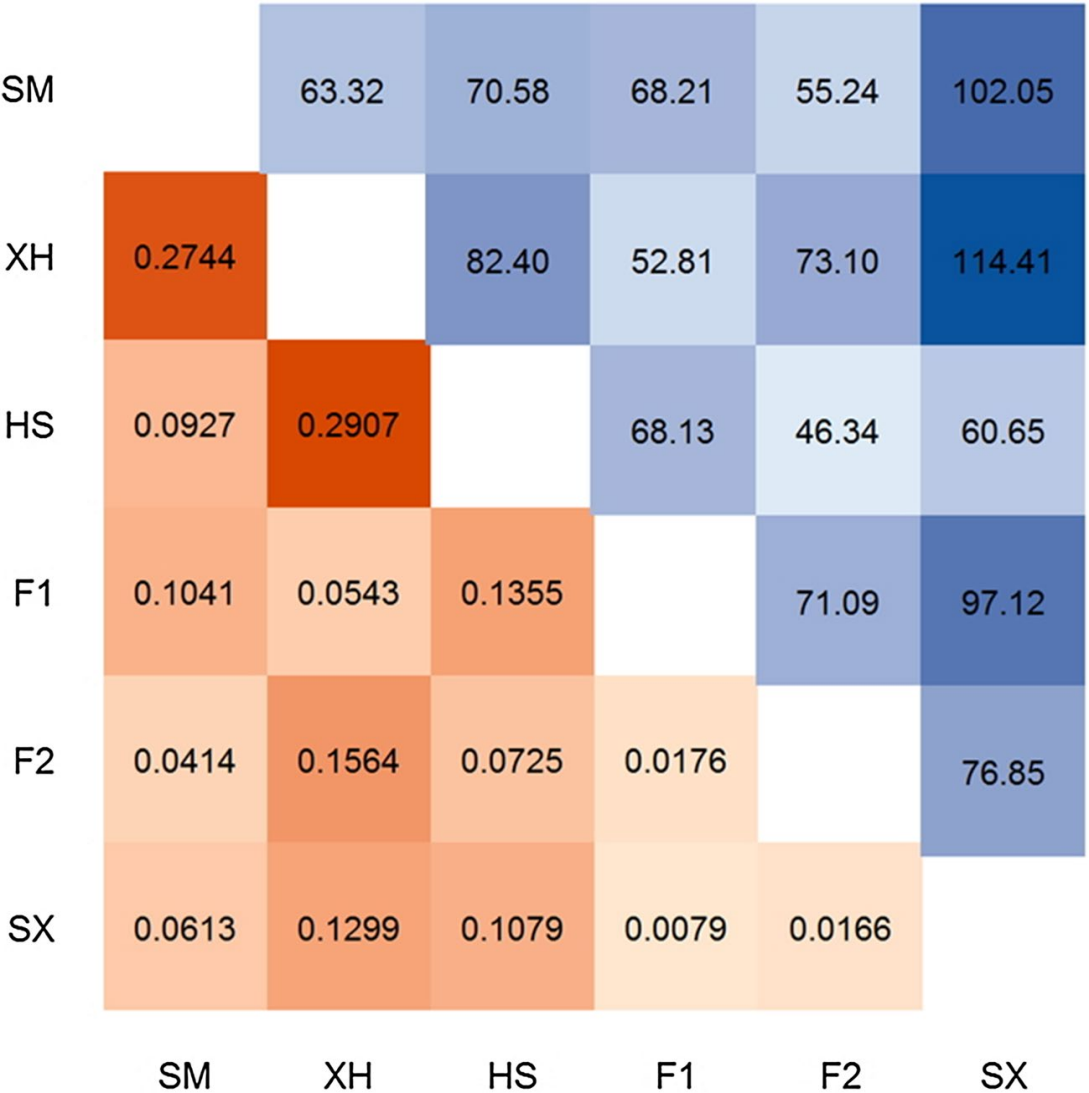
Genetic distances among populations. Pair-wise comparisons of six populations on Fst index calculated by all clean SNPs (lower-triangular, in orange) and mRNA expression-based Euclidean distances of 2866 DE genes (upper-triangular, in blue). Various shades of colours are proportional to values. (Color figure online)

Among all clean variants, we intend to detect population-specific or private genotype, which were defined as being exclusively found in one population. Accordingly, F1, F2 and Shuxuan populations were not found the population-specific variant. However, two, four and 58 specific genotypes were detected in Holstein, Simmental and Xuanhan populations, respectively (Supplementary Table 2). After this, the hierarchical clustering algorithm was also used for revealing individual relationships (Fig. 3a). Among the six populations, individuals from Xuanhan, Simmental and Holstein populations were robustly clustered together into three separate groups, whereas it couldn’t clearly distinguish individuals from F1, F2 and Xuanhan populations. We further surveyed Fst index among the six populations according to these SNPs (Fig. 4). The pair-wise Fst values of Xuanhan with Simmental and Holstein were significantly higher than others. By contrast, Fst values among F1, F2 and Shuxuan populations obviously decreased.

### Landscape of gene expressions

Up to 83.41% of all clean reads were successfully aligned to reference genome, and by which we totally assembled 119,157 transcripts of 33,246 genes. According to the definitions of transcript comparisons in Cuffcompare tool (Trapnell et al. 2012), 25.16% of our assembled transcripts matched exactly with the already annotated transcripts and 60.8% of them were also revealed as new isoforms to known genes (Supplementary Table 3). The mean and N50 (the middle sequence ordered by length) lengths of assembled transcripts were 2162 and 1377 bp, respectively.

Landscapes of gene expression were analyzed among the six populations according to the estimated ECF value. We first filtered out 17,254 genes with very low expression levels, which were defined as > 90% of samples having the estimated ECF lower than one. Subsequently, Euclidean distances among all samples were calculated by the normalized expression values; and by which individual relationships were demonstrated by hierarchical clustering algorithm (Fig. 3b). Among the six populations, only all samples of Simmental were clustered together, whereas individuals from other five populations were separately distributed.

### DE analysis among populations

**Fig. 5:**
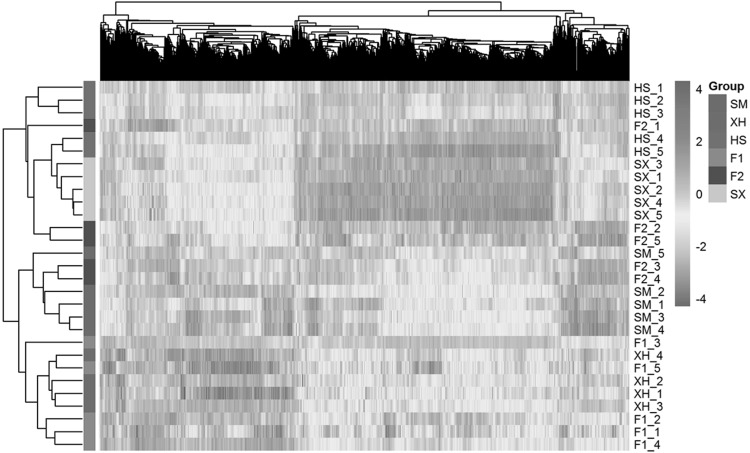
Biclustering of normalized expression levels for 2866 DE genes in all samples. Each row represents one sample from six populations (encoded by discrete colours), while the genes were individually demonstrated on columns. Hierarchical clustering patterns for both samples and genes were accordingly placed aside. The values of gene expression were log2 transformed, which were further illustrated by continuous colours from blue (low expression) to red (high expression). (Color figure online)

Subsequently, we employed linear model and determined the overall DE genes across six populations by the moderated F-statistic in combination with BH adjustment of multiple testing. With control of false discovery rate (FDR) < 0.01, a total of 2866 genes were supported to be differentially expressed. Based on these DE genes, a biclustering method was applied for revealing the underlying patterns of both gene expression and samples (Fig. 5). On the whole individuals from six populations were clustered together, which suggested higher intra-population similarity of gene expression. Meanwhile, a few clusters of coexpressed genes were also found within different populations. At population level, all 2866 DE genes were employed for calculating Euclidean distances according to normalized expression values (Fig. 4); and from which we revealed that the pairwise comparisons of Shuxuan with Xuanhan and Simmental had the highest dissimilarity.

**Fig. 6:**
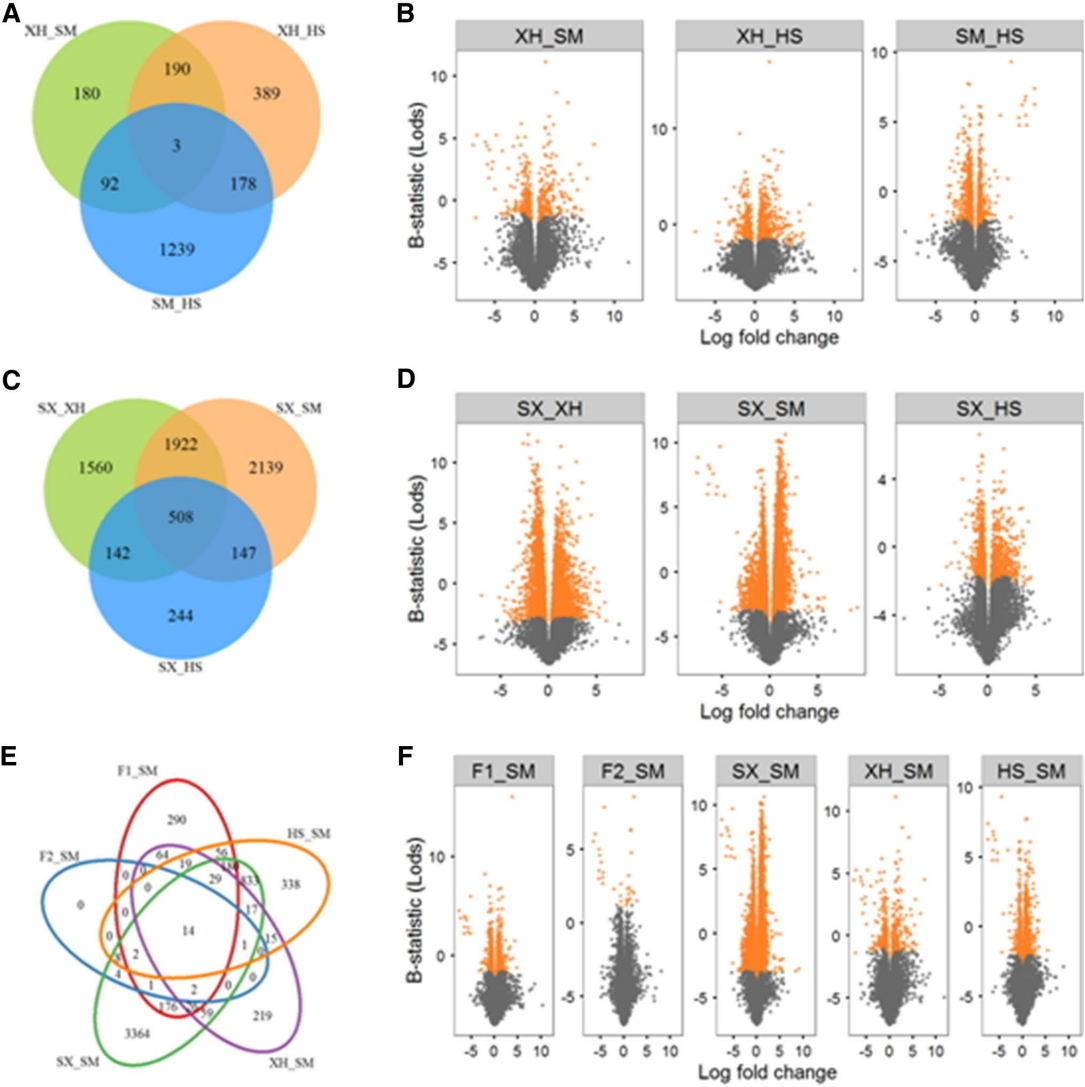
Venn and Volcano plots illustrate DE genes by pairwise comparisons of populations. First, the pairwise comparisons are conducted among Xuanhan, Simmental and Holstein (**a, b**). Second, Shuxuan is compared with Xuanhan, Simmental and Holstein one by one (**c, d**). Finally, the comparison between Simmental and other five populations are also analyzed (**e, f**). Venn plots show the numbers of shared and specific DE genes among different comparisons. Volcano plots demonstrate the DE genes with statistical significance (adjusted P value < 0.05, in orange). (Color figure online)

Subsequently, DE genes were also analyzed by pairwise comparisons of different populations (Fig. 6). Among the breeds of Xuanhan, Simmental and Holstein, up to 1239 DE genes were specifically detected between Simmental and Holstein, whereas other two comparisons resulted into less DE genes. As to the cultivated breed of Shuxuan, it was revealed to have more DE genes in comparison with both Xuanhan and Simmental than that i with Holstein. Among comparisons of Simmental with other five populations, we observed that there was much less DE genes in comparison with F1 and F2 populations; the comparison with Shuxuan, however, had the most DE genes.

## Discussion

In farm animals, discovery of polymorphic variants would be essential for the applications of marker assisted selection and conservation of genetic resources; and substantial progress has been gain in this field (Yang et al. 2013). In addition to whole-genome and whole-exome sequencing, several high-throughput sequencing techniques, such as the complexity reduction of polymorphic sequences and restriction-site-associated DNA sequencing, have been specifically proposed and applied to discovery of genomic-wide markers at low cost during the past years (Davey et al. 2011). However, most, if not all, of these methods always prefer to sequence random regions of genome. In addition to quantification of gene expression, RNA-Seq could also be used for exploring the variants exclusively being located within coding regions of genome, to which the preferable interesting would be paid for the biological investigations of interest. Therefore, Chepelev et al. first adopted RNA-Seq technology for identifying variants in transcribed regions of the human genome (Chepelev et al. 2009). Subsequently, specific bioinformatics pipelines or tools were proposed to facilitate variant discovery from RNA-Seq data (De Wit et al. 2012; Piskol et al. 2013; McKenna et al. 2010). In the present study, we successfully applied the RNA-Seq to study of population genetics in cattle simultaneously according to transcriptome-wide variants and gene expression. A large amount of variants were reliably detected with wide distribution among all chromosomes and could be used as genetic markers in future studies.

Although there are considerable morphological differences among various cattle breeds (Ajmone-Marsan et al. 2010), the genetic dissimilarity has also remained unknown at the genome-wide level especially for these Chinese indigenous breeds. It was first reported that a total of 2.44 million SNPs and 115,000 small InDels were detected by whole genome sequencing of a single *B. taurus* animal (Eck et al. 2009), which suggested the abundant resources of genetic polymorphism. Another landmark report was that the genome-wide SNPs were systematically compared among 497 cattle from 19 geographically and biologically diverse breeds; the results, however, supported that genetic diversity in cattle is as abundant as that in human although these cattle breeds had been subjected to intensively artificial selection (Gibbs et al. 2009). In the present study, we found that the individuals from different purebred breeds (Holstein, Simmental and Xuanhan) could be separately clustered according to 61,754 coding SNPs, which is not beyond the expectation because there breeds fairly distant in relation to their genetic origins. However, the same set of coding SNPs failed to distinguish these crossbreed populations of cattle involved in the present study. Therefore, we cautiously suggest that more attentions should be paid when selecting the marker panel for the individual assignment from crossbreed breeds (Wilkinson et al. 2011).

Because no more than five individuals were sampled from each population, we didn’t intend to detect the genome regions in relation to selective sweeps as similar to the prevalent analysis in former reports (Qanbari et al. 2014). By contrast, we alternatively analyzed the gene-wise differences on density of variant distribution and found that there was only a slight correlation between absolute and relative count of variants per gene. Furthermore, the density of variant distribution significantly differed among genes, which would suggest the differential biological functions and/or evolutionary processes. Of course, more attention should be paid to these genes that have abundant variants when performing association analysis of economic traits in cattle. Actually, a small set of function genes with known biological implications had been detected within genomic regions to be associated with extreme Fst values among different cattle breeds (Gibbs et al. 2009). However, our pairwise Fst values being estimated by coding SNPs were not well consistent with the expected relationships among populations in the present study, which would be resulted from the high background noise of transcriptome-wide SNPs (Qanbari et al. 2014).

In addition to genome-wide variants, the global gene expression could also be included into studies of population genetics and evolutionary biology. Brawand et al. (2011) first conducted evolutionary comparisons of transcriptome profiling among six organs of ten diverse mammalian species; and the result revealed divergent evolution rates among species, tissues and chromosomes. The comprehensive comparisons on transcriptional landscapes between human and mouse tissues were also reported (Lin et al. 2014). In the present study, we successfully assembled 15,992 informative genes with high reliability from RNA-Seq data among six cattle populations. Our systematic comparisons on gene expression revealed that there were obvious differences among populations, which was consistent with former report between Chinese Luxi and Angus beef cattle. However, the genetic structure of populations as being revealed by global gene expression couldn’t well support the known relationship of these populations in comparison with that deduced by transcriptome-wide variants. One possible reason is that the gene expression is more dynamic and would be easily affected by many known or unknown environmental factors. Here, we provided the basis for understanding landscape of differential expression among these six populations, and from which the detected DE genes could be subjected to further investigations for exploring biological implications, such as the divergent ability against heat stress between imported Holstein and indigenous Xuanhan cattle.

## Conclusion

In conclusion, we successfully employed RNA-Seq technology for revealing transcriptome-wide variants and gene expression among six Chinese cattle populations, and by which the genetic structures were comprehensively investigated. The results supported that SNPs would be better choice for elucidating the population structure of cattle than the quantification of gene expression. However, our study also provided landscape on the differentially expressed genes among these cattle populations, which would provide basis for future studies.

## Electronic supplementary material

Below is the link to the electronic supplementary material.

Supplementary material 1 (DOC 170 KB)
