# Supplementary material for: Genetic structure of six cattle populations revealed by transcriptome-wide SNPs and gene expression
Source: Genes Genomics. 2018 Mar 26;40(7):715–24. doi: 10.1007/s13258-018-0677-1 (PMC6015124; doi:10.1007/s13258-018-0677-1)

**Supplementary materials**

**Genetic structure of six cattle populations based on transcriptome-wide SNPs and gene expression**

Wei Wang^1^, Huai Wang^1^, Hui Tang^1^, Jia Gan^1^, Changgeng Shi^2^, Qing Lu^3^, Donghui Fang^1^, Jun Yi ^1, ¶^ and Maozhong Fu^1, ¶^

^1^ Sichuan Animal Science Academy, Animal Breeding and Genetics Key Laboratory of Sichuan Province, Chengdu, Sichuan 610066, P.R. China;

^2^ Xuanhan Animal Breeding and Improvement Station, Xuanhan County, Sichuan 636150, P.R. China;

^3^ Sichuan Yangping Cow Breeding Farm, Hongya County, Sichuan 620360 P.R. China.

^¶^ **Corresponding authors:**

Dr. Maozhong Fu; Tel: 86-028-84542298; Fax: 86-028-84542298; E-mail Address: fmz847464621@163.com

Dr. Jun Yi; Tel: 86-028-62561060; Fax: 86-028-62561060; E-mail Address: 372197981@QQ.com

**Supplementary Table 1**: Top 50 genes with the highest density of variants

| **Ensembl gene ids** | **Gene exon length (bp)** | **Relative counts of variants per kbp exons** | **Absolute counts of variants** |
| --- | --- | --- | --- |
| ENSBTAG00000047729 | 405 | 125.93 | 51 |
| ENSBTAG00000005711 | 558 | 82.44 | 46 |
| ENSBTAG00000010166 | 997 | 78.23 | 78 |
| ENSBTAG00000020116 | 1,431 | 62.19 | 89 |
| ENSBTAG00000046117 | 597 | 58.63 | 35 |
| ENSBTAG00000037937 | 4,308 | 57.10 | 246 |
| ENSBTAG00000002069 | 1,409 | 53.23 | 75 |
| ENSBTAG00000027204 | 1,281 | 47.62 | 61 |
| ENSBTAG00000007075 | 1,129 | 46.94 | 53 |
| ENSBTAG00000037605 | 857 | 44.34 | 38 |
| ENSBTAG00000039086 | 660 | 43.94 | 29 |
| ENSBTAG00000038494 | 1,023 | 43.01 | 44 |
| ENSBTAG00000005146 | 1,285 | 42.02 | 54 |
| ENSBTAG00000003743 | 1,059 | 41.55 | 44 |
| ENSBTAG00000045880 | 1,749 | 40.02 | 70 |
| ENSBTAG00000026437 | 831 | 39.71 | 33 |
| ENSBTAG00000045854 | 913 | 37.24 | 34 |
| ENSBTAG00000047449 | 610 | 36.07 | 22 |
| ENSBTAG00000045842 | 390 | 35.90 | 14 |
| ENSBTAG00000033034 | 461 | 34.71 | 16 |
| ENSBTAG00000004443 | 957 | 34.48 | 33 |
| ENSBTAG00000039413 | 858 | 33.80 | 29 |
| ENSBTAG00000038943 | 395 | 32.91 | 13 |
| ENSBTAG00000026068 | 518 | 32.82 | 17 |
| ENSBTAG00000038544 | 339 | 32.45 | 11 |
| ENSBTAG00000019588 | 1,184 | 32.09 | 38 |
| ENSBTAG00000031232 | 375 | 32.00 | 12 |
| ENSBTAG00000013055 | 813 | 31.98 | 26 |
| ENSBTAG00000038605 | 513 | 31.19 | 16 |
| ENSBTAG00000026944 | 1,539 | 31.19 | 48 |
| ENSBTAG00000039728 | 514 | 31.13 | 16 |
| ENSBTAG00000001308 | 2,410 | 31.12 | 75 |
| ENSBTAG00000013919 | 1,270 | 30.71 | 39 |
| ENSBTAG00000008328 | 762 | 30.18 | 23 |
| ENSBTAG00000039524 | 849 | 29.45 | 25 |
| ENSBTAG00000040261 | 345 | 28.99 | 10 |
| ENSBTAG00000000432 | 1,565 | 28.75 | 45 |
| ENSBTAG00000036224 | 1,959 | 28.59 | 56 |
| ENSBTAG00000037830 | 735 | 28.57 | 21 |
| ENSBTAG00000048184 | 1,273 | 28.28 | 36 |
| ENSBTAG00000038080 | 870 | 27.59 | 24 |
| ENSBTAG00000031160 | 1,442 | 27.05 | 39 |
| ENSBTAG00000047193 | 336 | 26.79 | 9 |
| ENSBTAG00000034302 | 972 | 26.75 | 26 |
| ENSBTAG00000039956 | 427 | 25.76 | 11 |
| ENSBTAG00000004318 | 798 | 25.06 | 20 |
| ENSBTAG00000009656 | 1,016 | 24.61 | 25 |
| ENSBTAG00000048145 | 695 | 24.46 | 17 |
| ENSBTAG00000039691 | 904 | 24.34 | 22 |
| ENSBTAG00000046660 | 370 | 24.32 | 9 |
| **Mean** | **979.12** | **38.56** | **38.46** |

Note: the genes shorter than 300 bp in length were not included for calculating relative count of variants

**Supplementary Table 2**: Population specific variants and genotypes

| **Populations** | **Chromosomes and positions** | **Private genotypes** | **Common genotypes** |
| --- | --- | --- | --- |
| Holstein | chr1: 145871621 | CC | CT, TT |
|  | chr15: 82125280 | TT | CT, CC |
| Simmental | chr10: 1119957 | AG | AA |
|  | chr10: 1119960 | CG | GG |
|  | chr12: 36619659 | TT | CT, CC |
|  | chr12: 36700958 | TT | CT, CC |
| Xuanhan | chr1: 65928573 | GA | GG |
|  | chr1: 65928591 | AC | AA |
|  | chr1: 68048694 | AG | AA |
|  | chr1: 136860046 | CC | TT, CT |
|  | chr2: 71424267 | GG | AA, AG |
|  | chr2: 71496624 | TT | CC, CT |
|  | chr3: 16521395 | CC | TT, TC |
|  | chr3: 16560954 | GG | CC, CG |
|  | chr5: 30867162 | CC | TT, TC |
|  | chr5: 30867971 | GG | CC, CG |
|  | chr5: 109436490 | GG | AA, AG |
|  | chr5: 109454377 | GG | AA, AG |
|  | chr5: 109565934 | AA | GA |
|  | chr7: 17868620 | GG | TG, TT |
|  | chr7: 45600313 | CC | GG, GC |
|  | chr9: 18755547 | AA | CC, CA |
|  | chr9: 96602023 | TT | GG, GT |
|  | chr10: 102885618 | GG | CC, CG |
|  | chr11: 2725460 | CC | TT, CT |
|  | chr13: 54073380 | CC | TT, CT |
|  | chr13: 54077431 | TT | CC, CT |
|  | chr13: 54077432 | GG | AA, AG |
|  | chr13: 54077512 | TT | AA, AT |
|  | chr13: 54077570 | TT | GG, GT |
|  | chr13: 54077619 | TT | CC, CT |
|  | chr13: 54077626 | GG | TG, TT |
|  | chr13: 54078248 | TT | CC, CT |
|  | chr13: 54078326 | AA | GG, GA |
|  | chr13: 54078341 | AA | GG, GA |
|  | chr13: 54078364 | CC | TT, TC |
|  | chr13: 54078765 | CC | GG, GC |
|  | chr13: 54124488 | TT | CC, TC |
|  | chr13: 73467839 | TG | TT |
|  | chr13: 80578783 | GA | GG |
|  | chr16: 2765105 | GG | AA, AG |
|  | chr16: 2765861 | AA | CC, CA |
|  | chr16: 3575726 | GG | AA, AG |
|  | chr16: 50476685 | AA | GG, GA |
|  | chr16: 50510621 | TT | CC, CT |
|  | chr16: 50682944 | TT | CC, CT |
|  | chr16: 50683434 | AA | GG, GA |
|  | chr16: 50690323 | AA | GG, GA |
|  | chr16: 51687771 | AA | TT, TA |
|  | chr17: 74561512 | GG | AA, AG |
|  | chr17: 75001505 | CC | TT, TC |
|  | chr19: 59071613 | GG | AA, AG |
|  | chr22: 32488091 | TT | CC, CT |
|  | chr22: 54041501 | GG | AA, AG |
|  | chr22: 54041888 | CC | AA, AC |
|  | chr22: 54041980 | CC | AA, AC |
|  | chr25: 35991910 | TT | GG, TG |
|  | chr26: 9370330 | GG | AA, AG |
|  | chr26: 9377498 | CC | TT, TC |
|  | chr27: 38015426 | GG | AA, AG |
|  | chr27: 38015494 | GG | AA, AG |
|  | chr29: 44459687 | CC | TT, TC |
|  | chr29: 44479352 | CC | TT, TC |
|  | chr29: 44503834 | GG | AA, AG |

**Supplementary Table 3:** Comparisons of the assembled transcripts with already annotated genes in reference genome

| **Count of assembled transcripts** | **Class codes of comparison** | **Official description of class codes** |
| --- | --- | --- |
| 29,975 (25.2%) | = | Complete match of intron chain |
| 89 (< 0.1%) | s | An intron of the transfrag overlaps a reference intron on the opposite strand |
| 72,458 (60.8%) | j | Potentially novel isoform |
| 12,452 (10.5%) | u | Unknown, intergenic transcript |
| 17 (< 0.1%) | i | A transfrag falling entirely within a reference intron |
| 1,937 (1.6%) | o | Generic exonic overlap with a reference transcript |
| 2,228 (1.9%) | x | Exonic overlap with reference on the opposite strand |
| 1 (< 0.1%) | p | Possible polymerase run-on fragment |

**Supplementary Figure 1** **Outline of Xushuan cattle breeding process and genetic relationships among six populations sampled in the present study**. The colourful pie charts represent different breeds, in which the proportion of genetic components were further shown by breed-specific colours.


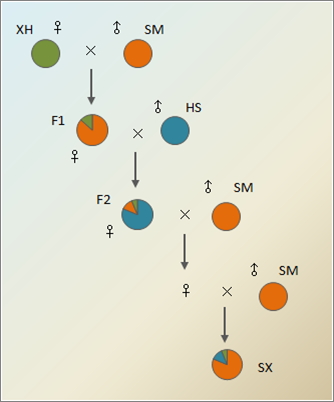

Supplement: Supplementary file 1 — Supplementary material 1 (DOC 170 KB) [file 13258_2018_677_MOESM1_ESM.doc]
